# Dense fluidized granular media in microgravity

**DOI:** 10.1038/s41526-017-0030-z

**Published:** 2017-11-03

**Authors:** Philip Born, Johannes Schmitz, Matthias Sperl

**Affiliations:** 1 Institut für Materialphysik im Weltraum, Deutsches Zentrum für Luft- und Raumfahrt (DLR), 51170 Köln, Germany; 2 Institut für Theoretische Physik, Universität zu Köln, 50937 Köln, Germany

## Abstract

Handling and transport of granular media are inevitably governed by the settling of particles. Settling into a dense state is one of the defining characteristics of granular media, among dissipation and absence of thermal agitation. Hence, settling complicates the adaptation of microscopic theories from atomic, molecular, or colloidal media to granular media. It is desirable to provide experiments in which selectively one of the granular characteristics is tuned to test suitable adaptation of a theory. Here we show that gas fluidization of granular media in microgravity is a suitable approach to achieve steady states closer to thermally agitated systems free of settling. We use diffusing-wave spectroscopy to compare the spatial homogeneity and the microscopic dynamics of gas-fluidized granular media on the ground and in drop tower flights with increasing packing densities up to full arrest. The gas fluidization on the ground leads to inhomogeneous states as known from fluidized beds, and partial arrest occurs at packing fractions lower than the full arrested packing. The granular medium in microgravity in contrast attains a homogeneous state with complete mobilization even close to full arrest. Fluidized granular media thus can be studied in microgravity with dynamics and packing fractions not achievable on the ground.

## Granular matter: Coming to rest in low gravity

Microgravity provides a better environment to study how grains move when agitated, scientists in Germany show. Philip Born and co-workers from the Institute of Materials Physics in Space demonstrate that microgravity analogue conditions enable studies on particle dynamics at packing densities not achievable on the ground. Dense granular media under external agitation tends to partially arrest on the ground due to particle collision and settling caused by gravity. This diverseness of the medium makes it hard to develop a theory that can describe the general behavior. Born and colleagues use diffusing-wave spectroscopy to compare the spatial homogeneity and microscopic dynamics of granular media on the ground and in drop towers. They show that unlike on the ground, granular media in microgravity conditions can reach a homogeneous state without partial arrest at high packing densities.

## Introduction

Gravitational settling is one of the defining characteristics of granular media, among dissipative collisions and the absence of thermal agitation. The interplay of these effects with the required agitation leads to complex fluidized states of granular media. Even simple harmonic shaking of granular media gives rise to a variety of states,^1,2^ which cannot be described by present microscopic theories.^3–5^ Selective tuning of the characteristics of granular media thus is desirable to better understand the connection among microscopic theories like kinetic theory or mode-coupling theory and experimental investigations.

Here, we present a study on dense fluidized granular media with and without gravitational settling. A central question is, whether fluidized granular media take steady states in microgravity, which are close to states of media in thermal equilibrium. A fluidization provides agitation to reach a dynamic steady state of a granular medium. A thermalization, which can be compared to media in thermal equilibrium, is not necessarily reached in such a steady state. A thermalization requires normal distribution of uncorrelated velocities and a homogeneous particle number density in the steady state.^6–8^ A normal distribution of uncorrelated particle velocities requires short agitation timescales compared to the timescales of dissipative collisions of the particles in the medium.^6–8^ Small ratios of agitation timescales to collision timescales are experimentally accessible in two-dimensional systems, where particles can be individually agitated by air-hockey-like or vibrating tables.^9–11^ Application of the same agitation approaches to dense three-dimensional media, where particles collide much more frequently with each other than with the container walls, leads to very short timescales of dissipative collisions and the thermalization fails.^7^ This situation of too large agitation timescales is characterized by enhanced fluctuations of the local number density or clustering of the granular medium.^8^

An approach towards short agitation timescales compared to the timescales of dissipative collisions in bulk granular media is agitation in a fluidized bed working with a gas or a liquid. In principle, fluidization by gas or a liquid allows for more homogeneous agitation than agitation from the boundaries, since each particle in the granular medium can take up energy from the fluid stream. This benefit comes at the cost of hydrodynamic interactions among the particles especially with liquid-fluidized beds^12,13^ and a hydrodynamically instable steady state prone to fluctuations in the local particle number density^12,14^ and in particle dynamics.^15^ Importantly, the confining stresses imposed on fluidized beds by gravitation induce a coupling between agitation strength and packing fraction in fluidized beds.^12,13^ The packing fraction depends linearly on the fluid velocity over large ranges of fluid velocities, and steady states at high packing fractions can consequently only be reached at low agitation strengths. The gravitational confining stresses finally impose a static state at high packing fractions or low fluid velocities close to the fully settled state. This static state is partially stabilized by permanent particle contacts and partially by the fluid flow.^12,13,16,17^ These effects limit the applicability of fluidized beds for investigations on dense granular media on the ground. Liquid-fluidized beds circumvent some of the problems by partial density matching of the particles, but bear the problem of the strong hydrodynamic interactions and viscous damping.

Fluidizing granular media without gravitational settling consequently may allow for dynamic steady states of dense granular media close to media in thermal equilibrium. Granular media should become fluidizable at any packing fraction with nearly arbitrary agitation strength, as confining stresses vanish and only particle cohesion must be overcome by the fluid to agitate the particles.^18^ The packing fraction consequently becomes a parameter, which could be externally controlled by the volume of the container of the medium.

We test this aspect with gas-fluidizing granular particles in the microgravity environment achieved during drop tower flights. We compare the parameters filling fraction of a sample cell Φ, which is the ratio of the volume of the sample to the volume of the sample cell, the packing fraction of the granular medium *ϕ*, as can be observed from the expansion of the granular medium, and the local packing fraction *ϕ*_loc_, which is the packing fraction in certain subvolumes of the sample. In a thermalized dynamic steady state, with fast enough agitation compared to dissipation, all the three parameters should be equal.

We apply an evaluation methodology for diffusing-wave spectroscopy (DWS) recently presented to classify homogeneity and degree of fluidization within the packing.^14^ We give a brief account of the setup for fluidizing granular particles in drop tower flights and on the ground and summarize the light scattering methodology in the section Methods at the end of the manuscript. In the following section Results, we compare the achieved fluidization by means of the DWS measurements. We discuss in section Discussion that fluidization in microgravity differs from fluidization on the ground. The packing fraction *ϕ* of the fluidized granular sample stays higher than the filling fraction of the sample cell Φ on the ground at low-filling fractions Φ, while *ϕ* approximates Φ in microgravity in this situation. Increasing the filling fraction Φ beyond the packing fraction *ϕ* reached by the fluidized granular medium at lower filling Φ leads to a partially arrested sample on the ground and homogeneous fluidization fails. In contrast, the sample indeed stays homogeneously fluidized throughout the packing in microgravity, even close to the packing fraction of full arrest. Microscopic dynamics are observed to be always slower in microgravity than on the ground. Concludingly, fluidization in microgravity thus leads to a less complex state, which may provide the basis for testing microscopic theories for granular media.

## Results

**Fig. 1:**
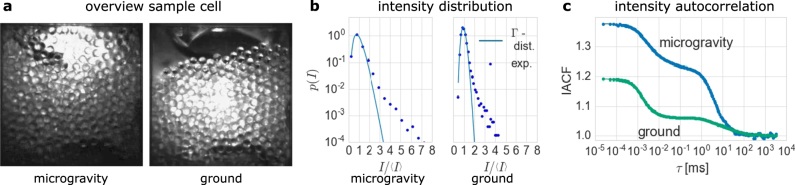
Comparison of the fluidized granular medium in microgravity and on the ground at a low- filling fraction Φ of 0.48. The medium expands in microgravity and particles are distributed throughout the sample cell, which equilibrates filling fraction Φ and packing fraction *ϕ*. On the ground, the medium stays settled at a high-packing fraction *ϕ* (**a)**. Larger voids move through the sample in both cases, thus local packing fraction fluctuations occur which lead to strong measured deviations from Γ-distributed intensity values in both cases (**b)**. The obtained intensity autocorrelation functions (IACF) accordingly exhibit two distinct decays (**c**)

The fluidization of the granular medium behaves at the lowest filling fraction Φ as described in the Introduction. The particles are agitated by the gas stream and the packing expands on the ground, but remain settled with a free surface due to the gravitational confining pressure (Fig. 1a). The packing fraction *ϕ* of the fluidized state on the ground can be estimated from the height of the packing in the overview camera image to be ≈0.63, close to the limit of random close packing at *ϕ* ≈ 0.64. In contrast, the granular medium expands to explore the whole available volume for the same gas flow in microgravity (µg) (Fig. 1a). The filling fraction Φ and the packing fraction *ϕ* within the granular medium thus seems to be equal in μg.

The intensity distributions obtained from the fast count rate traces of the used hardware correlator show deviations from Γ-distributions in both cases, which indicates the presence of temporal fluctuations of *ϕ*_loc_ in the probed volume (Fig. 1b). The deviation from a fitted Γ-distribution is larger in microgravity than on the ground. The IACFs consequently exhibit two distinct decays in correlation, originating from the microscopic particle displacements and the packing fraction fluctuations (Fig. 1c). The amplitude of the second decay is smaller on the ground than in μg, leading to a smaller total amplitude of the IACF on the ground.

**Fig. 2:**
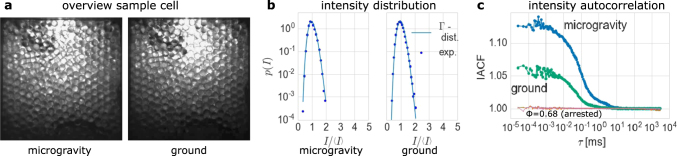
Fluidization of the granular medium at a high-filling fraction Φ = 0.679. The particles now fill the whole sample cell and hexagonal order can be observed on the ground and in microgravity (**a**). The distributions of the measured transmitted intensities follow Γ-distribution, which are predicted for intensity fluctuations emerging from random phase shifts (**b**). The IACFs accordingly decay in a single, nearly exponential decay, with no measurable contributions from local packing fraction fluctuations (**c**). Although the samples are forced to take the same filling fraction Φ and packing fraction *ϕ* in both the cases, the IACFs do not superpose. The amplitude $\tilde E$ of the IACF on the ground is only half as high as the amplitude measured in microgravity. Also shown in **c** are the autocorrelation functions measured samples at Φ = 0.68, which is achieved with a minute compression by the piston of 20 μm compared to Φ = 0.679. No decay in correlation can be observed, thus the samples become fully arrested

The situation considerably changes at high-filling fractions Φ (Fig. 2). The volume of the sample cell can be lowered down to a full arrest of the particles. At a filling fraction of Φ = 0.68 intensity fluctuations cease and the intensity autocorrelation does not show any decay anymore when the sample is compressed by the piston. But a minute withdrawal of the piston of 20 μm, a 25th of a particle diameter and a reduction in filling fraction by ΔΦ = 0.001, allows agitation of the particles. The intensity distributions follow Γ-distributions (Fig. 2b), indicating the absence of fluctuations of local number densities *ϕ*_loc_ in time. The IACF accordingly exhibits only a single decay from the field fluctuations alone (Fig. 2c). Again, the amplitude is smaller on the ground.

**Fig. 3:**
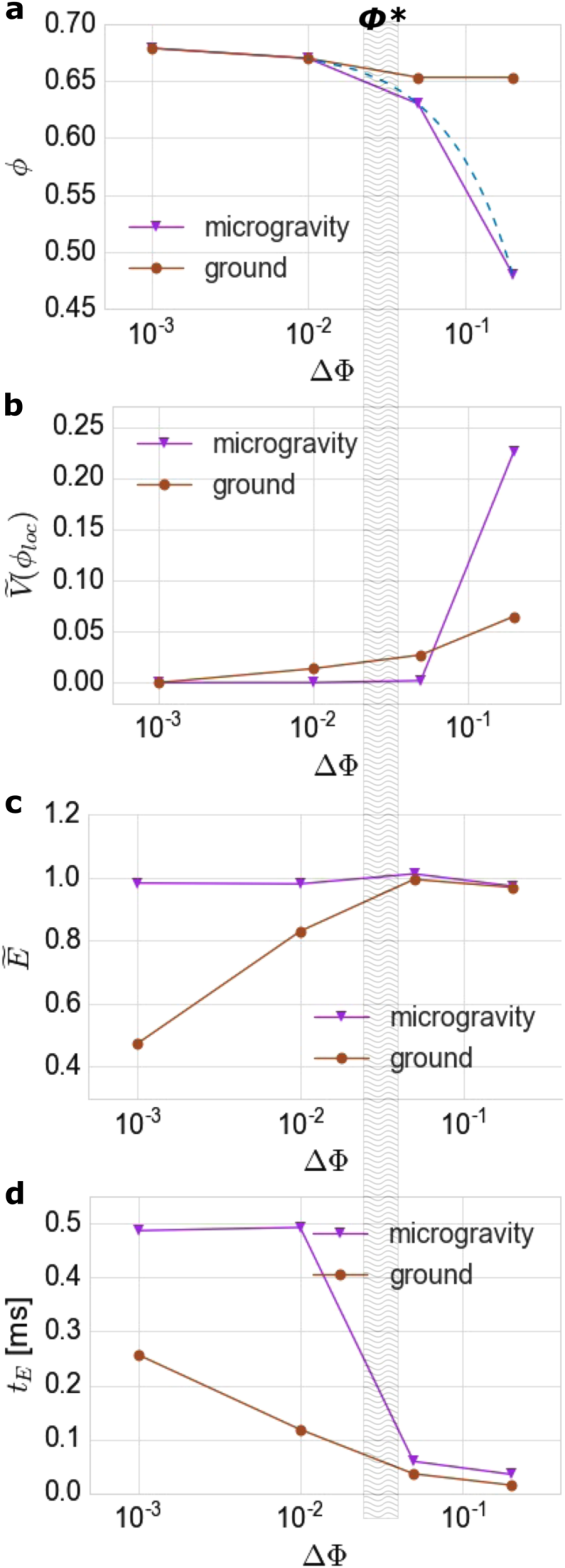
Summary of the fluidization experiments on the ground and in μg. The parameters in **b**–**d** are obtained from exponential fits to the measured autocorrelation functions, the lines are guidelines to the eye. The parameters are given as a function of the difference ΔΦ of Φ from full arrest at filling fraction 0.68. **a** The packing fraction *ϕ* obtained from the overview images. The dashed line gives the expectation *ϕ* = Φ. **b** The variance of the total transmitted intensity $\tilde V(\phi _{{\mathrm{loc}}})$, indicating fluctuations of local number densities. **c** The amplitude of the field autocorrelation $\tilde E$, indicating the degree of fluidization. **d** The decay time *t*_E_ the field autocorrelation, indicating the kinetic energy of the particles. The dashed region Φ^*^ ≈ 0.63…0.64 marks the filling fraction corresponding to random close packing and to the packing fraction the fluidized medium on the ground at large ΔΦ. See text for further discussion

The transition between these two opposites is summarized in Fig. 3. The isolated contributions by the field autocorrelation and by the autocorrelation of the total transmitted intensity are shown, which are sensitive to different aspects of the fluidized granular sample (see section Methods).^14^ The isolated field autocorrelation functions exhibit an amplitude close to 1 after correcting with the *β*-factor of 3 (see section Methods), indicating normal distribution of the field values. We fit the obtained correlation functions with exponential decays to get a clearer view on the distinct evolution of the dynamics on the ground and in μg. The decay time *t*_E_ and the normalized amplitude $\tilde E$ of the field autocorrelation function and the variance $\tilde V(\phi _{{\mathrm{loc}}})$ of the total transmitted intensities as obtained from the fits are given in Fig. 3 as function of the distance to the filling fraction of full arrest ΔΦ. The packing fraction *ϕ* as obtained from the overview camera is given in addition.

The observables share common trends on the ground and in the microgravity with increasing ΔΦ. At small ΔΦ both samples take *ϕ* = Φ and the packing fraction *ϕ* lowers with ΔΦ. The variance of the total transmitted intensity $\tilde V(\phi _{{\mathrm{loc}}})$ increases with ΔΦ. The amplitude $\tilde E$ approaches 1 at large ΔΦ. The decay time decreases with ΔΦ. And in both cases the strongest changes happen around a filling fraction corresponding to the random close packing fraction.

While this general behavior is the same on the ground as in μg, there are differences in the details. These details will be discussed in the following section, they allow to deduce a distinct evolution of the fluidized sample on the ground and in microgravity.

## Discussion

Fluidization of granular media in microgravity might become interesting for the possibility of realizing a thermalized state of densely packed hard particles. This possibility was tested by DWS, which allows to probe the homogeneity of an agitated granular medium and the microscopic particle dynamics. In Fig. 3 differences among the fluidized states on the ground and in μg become obvious. Notably, the results indicate that states close to thermalization were reached in μg:

First, no differences among the packing fraction observable with the overview camera *ϕ* and the filling fraction defined by the sample cell volume Φ are observable in μg, the granular medium expands to explore the whole sample cell. on the ground the sample stays sedimented in a dense state, and the apparent packing fraction *ϕ* becomes independent of filling fraction Φ at large ΔΦ (Fig. 3a).

Second, the variance of the total transmitted intensity $\tilde V(\phi _{{\mathrm{loc}}})$ indicates the amplitude of temporal fluctuations of the local packing fraction *ϕ*_loc_ in the probed volume. The measured variances $\tilde V(\phi _{{\mathrm{loc}}})$ thus show a different evolution of the homogeneity of the fluidized medium on the ground and in microgravity. At the largest ΔΦ, stronger fluctuations are present in μg than on the ground, as can be seen from the higher $\tilde V(\phi _{{\mathrm{loc}}})$. The confining pressure by gravitation prevents unbounded growth of air bubbles and voids in the granular medium.^12^ In microgravity, this stabilization fails, and larger voids can form in the medium. However, these fluctuations cease faster below detectability in *μ*g than on the ground with reduction of ΔΦ, where a small non-vanishing $\tilde V(\phi _{{\mathrm{loc}}})$ indicates an inhomogeneous, fluctuating *ϕ*_loc_ even at small ΔΦ (Fig. 3b).

Third, the development of the field autocorrelation functions with ΔΦ indicate different particle dynamics on the ground and in μg (Figs. 3c, d). The amplitude of the field autocorrelation $\tilde E$ is determined by fluctuations of the phases in the scattered light, and a reduction of $\tilde E$ indicates a partially static sample. The decay time *t*_E_ represents the rotational and translation velocities of the particles, thus is an indicator of the kinetic energy of the particles. As the kinetic energy is determined by the dissipative collisions for a given agitation,^8^ increases in the decay time *t*_E_ are also connected to an increasing packing fraction. At the largest ΔΦ the microscopic dynamics result in similar *t*_E_ and $\tilde E$ on the ground and in microgravity (Fig. 3c, d), notwithstanding the differences in macroscopic homogeneity as indicated by the total transmitted intensity autocorrelation (Fig. 3b). Particles thus have similar translational and rotational velocities on the ground and in μg at this ΔΦ. This changes when the sample is compressed beyond the packing fraction of the fluidized medium on the ground and the random close packing fraction Φ^*^ (Fig. 3c, d). Although in this situation the sample on the ground and the sample in *μ*g are forced to take the same global packing fraction *ϕ* = Φ, the microscopic dynamics turn out to be different. The amplitude of field autocorrelation $\tilde E$ decreases once the piston starts compressing the fluidized granular medium on the ground, while the decay time *t*_E_ increases, but not as much as in μg (Fig. 3c, d). The reduction of $\tilde E$ indicates a static component in the transmitted light, or in other terms, parts of the granular medium become arrested. The decay times *t*_E_ do not change dramatically upon compression onset, which indicates that the particles in the remaining fluidized regions of the sample nearly have the same kinetic energy as at large ΔΦ. The amplitude of the field correlation $\tilde E$ in contrast does not vary upon compression and lowering ΔΦ in μg (Fig. 3c). The granular particles thus stay mobile throughout the probed volume. However, the decay time *t*_E_ of the autocorrelation exhibits a strong increase when Φ increases beyond 0.64, equivalent of random close-packing fraction (Fig. 3d). This increase in packing fraction is only possible with local crystalline packing of the particles, as can also be observed in the side view of the packing (see Fig. 2). The increased collision rates in the high-density polycrystalline state cause intensified dissipation and a reduced *t*_E_.

**Fig. 4:**
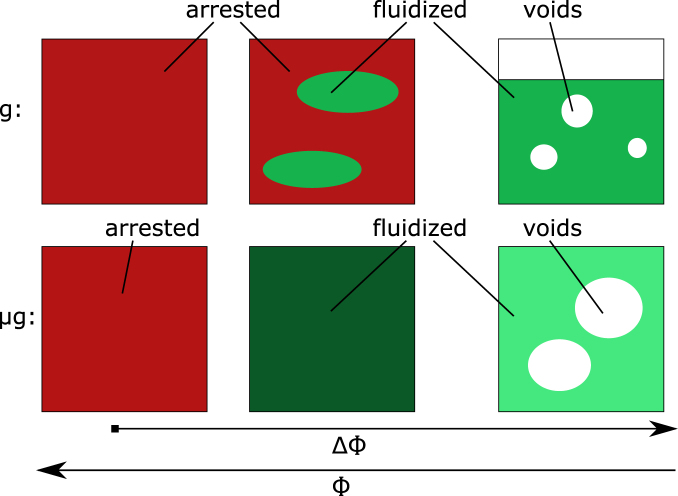
Schematic drawing of the different evolution of fluidized granular media with increasing the filling fraction Φ on the ground (*g*) and in microgravity (μg) based on the DWS measurements. The samples become fully fluidized in both cases at low-filling fraction. The sample stays settled with moderate packing fraction and a free surface on the ground (intermediate green area), while it expands in microgravity to fill the cell with a low packing fraction (light green area). This gives space for the formation and motion of larger voids in the sample than on the ground (white areas). An increase of Φ close to the limit of full arrest leads to a homogeneous fluidized state with high packing fraction in microgravity (dark green area). On the ground, the sample separates into steady fluidized regions where the packing fraction of the fully fluidized state is locally preserved (intermediate green areas), and into arrested densely packed regions (red area). Further increase in packing fraction leads to fully arrested samples in both cases (red area, ΔΦ = 0)

The different evolution of granular media fluidized on the ground and in microgravity with increasing the filling fraction as deduced from the DWS measurements are summarized in Fig. 4. The samples become fully fluidized at large ΔΦ in both cases. Still, the fluidized sample stays settled at packing fractions *ϕ* close to random close packing on the ground. Increasing the filling fraction Φ beyond the packing fraction of the fluidized sample leads to a heterogeneous sample on the ground: Parts of the sample get arrested, presumably at a high local packing fraction *ϕ*_loc_ of full arrest. The particles in the remaining parts of the sample can achieve similar kinetic energies as at lower filling fractions Φ as the decay times *t*_E_ stay similar, thus the local packing fractions *ϕ*_loc_ stay close to the unperturbed packing fraction of the sample fluidized at lower filling fractions Φ.

In contrast, the sample expands to fill the whole sample cell in μg and Φ = *ϕ*. When ΔΦ is lowered beyond random close packing close towards full arrest, the sample takes a homogeneous polycrystalline state. In this homogeneous state presumably Φ = *ϕ* = *ϕ*_loc_. The observed larger decay times in microgravity indicate stronger dissipation and a higher packing fraction than in the remaining fluidized regions at the same filling fraction on the ground.

The results indicate that samples on the ground and in microgravity approach full arrest along two fundamentally different pathways (Fig. 4). A fluidized sample on the ground reacts to compression with increasing fractions of the sample being arrested until the whole sample is arrested. The remaining fractions of the sample keep the low packing fraction and the fast dynamics of the fluidized state. In microgravity, a fluidized sample reacts to compression with a homogeneous slowing down of dynamics, until the whole sample is arrested.

A full thermalization, as defined by a normal velocity distribution in three dimensions,^7^ is not yet proven by our results. The nearly exponential decay of the field autocorrelation might indicate a diffusion-like motion of scattering objects within the sample.^19^ The uncertainty on the nature of the scattering processes in a granular sample^14^ prevents deduction of the statistics of particle motion from the results at this stage. Still, prerequisites for a thermalization can be established close to the limit of full arrest. Agitation time scales are short enough to prevent dissipative clustering and a heterogeneous packing fraction, and homogeneous agitation of the particles can be reached.

Two remarks may exemplify the relevance of the results. First, the small length scales involved in the presented experiments set constraints for future experiments. The measurements at the two highest volume fractions are separated only by a piston motion of 20 μm, or a difference in filling fraction ΔΦ of 0.001. Despite the minute difference, the major changes of the correlation functions and thus in the dynamics of the particles happen between the fully arrested state and the next lower packing fraction. The decay times *t*_E_ of the field correlation reduce from infinity to tenths of milliseconds and the amplitude $\tilde E$ increases from 0 to 1 within this change in Φ of 0.001. This has implications for the level of control required for investigations on dynamics close to jamming, for example, using vibration fluidization. It must be ensured that the surfaces vibrating to agitate the sample do not impose even minute changes to the volume of the sample cell to prevent temporarily arresting the sample and suppression of any dynamics. This suggests to avoid usage of single oscillating pistons or counterpropagating periodically oscillating sample cell walls, as are used for studies at lower volume fractions,^20^ for future studies at high packing fractions.

Second, the differences among fluidization on ground and in microgravity may be compared to the phenomenological Geldart classification of fluidized granular media on ground.^21^ The sample used in the experiments here clearly belongs the class of bubbling granular media. The particles are macroscopic and are suspended in air, and the bubbling becomes obvious from the density fluctuations at large ΔΦ on ground and in microgravity. A fluidized bed with a homogeneous macroscopic appearance can be achieved on ground by using much smaller particles or denser fluids to achieve the Geldart regime of aeratable particles. However, the aeratable regime is characterized by strong hydrodynamic interactions or particle cohesion, leading to a clustered or aggregated microscopic structure of the fluidized granular medium and a strong expansion of the medium before fluidization.^13^ The homogeneous state of the granular medium fluidized in microgravity is distinct from this aeratable Geldart regime and resembles a unique new regime. The particles are still large and much denser than the fluid, thus the role of particle collisions is more prominent than for aeratable particles, and fluidization is achieved even at high packing fraction close to full arrest, where aeratable granular media have to expand first before fluidization.

In conclusion, we present a comparative study on the fluidization of granular media in microgravity and on the ground. On the ground, different local densities with static and agitated particles coexist at higher filling fractions. The fluidized state in μg turns out to be characterizable by a single packing fraction and homogeneous dynamics. Microgravity thus allows for the creation of well-fluidized states, which are impossible to achieve on the ground and likely closer to the homogeneous states assumed in microscopic theories.

## Methods

**Fig. 5:**
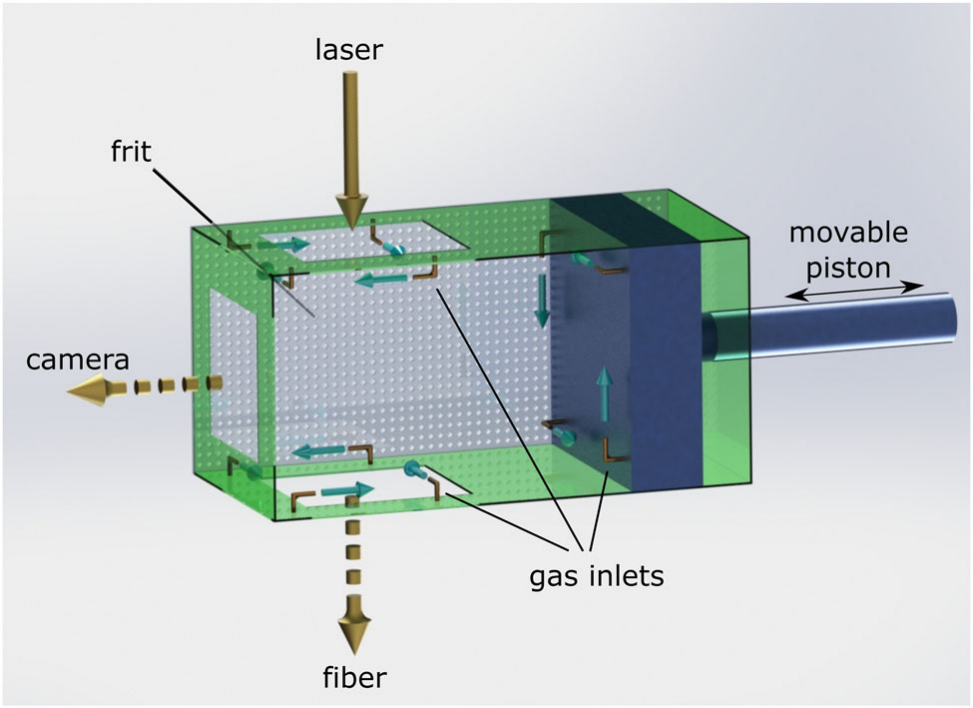
CAD-drawing of the sample cell used in the experiments. The sample cell had a cross section of 1 × 1 cm^2^ and a length adjustable by a motor-driven piston between 1 and 2 cm. Gas entered the cell through 12 inlets in the top and bottom windows and the piston to agitate the particles. The sample was illuminated by a 532 nm laser through the top window, and was investigated either by an overview camera through the side window or with a fiber probe through the bottom window

The granular sample was agitated by pressing a nitrogen flow with controlled humidity of 75%RH and a fixed flow rate of 2 l/min through the packing of the particles. The flow rate was chosen to be above the fluidization threshold and in a range where a double decay in the intensity autocorrelation functions is observed on the ground, but below the threshold of entrainment.^18,22^ The gas was directed through 12 gas inlets into the sample cell and left through the sidewalls of the cell consisting of glass frits (see Fig. 5). The gas inlets were aligned to direct the gas tangentially to the cell walls, in such a way that four inlets form a vortex, respectively, counter-rotating to the opposite and adjacent vortex and ensuring collisional motion of the particles. The sample cell volume was adjustable by a piston. The cell allowed for DWS measurements in transmission and for observation with an overview camera from the side. More details of the setup and of the microgravity experiments are described in a previous study.^22^ Here we used 961.86 mm^3^ of 500 μm monodisperse polystyrene spheres (Microbeads AS, Norway) as granular medium, which were additionally treated with hydrophobic aerosil particles (Evonik, Germany) to minimize cohesion and friction.

We set the volume of the sample cell in between the drop tower flights to adjust the filling fraction from Φ = 0.68, where full particle arrest was achieved, to Φ = 0.679, 0.67, 0.63 and 0.48 (or ΔΦ = 0, 0.001, 0.01, 0.05, and 0.2), to test whether the packing fraction can be defined independently of agitation. The highest filling fraction was achieved by simultaneously agitating the particles and repeatedly compressing with the piston, until no further compaction was detectable. The high achievable filling fraction indicates local hexagonal packing of the monodisperse particles, which could be confirmed by the overview camera.

The microgravity (μg) required for the experiments was achieved during drop tower flights at the ZARM drop tower in Bremen, Germany. The catapult device of the drop tower facilitated 9 s of microgravity with rest accelerations of below 10^−6^ g.

The reaction of the granular medium to the gas agitation was characterized by the intensity autocorrelation function (IACF) obtained from a hardware correlator in the DWS measurements. The intensity fluctuations and the IACF were measured in the drop tower flights for 8 s, after 1 s of waiting time for cessation of effects of the catapult start and for reaching a steady state. Subsequently, IACFs were also recorded on the ground without changes for 100 s.

The light intensity *I*(*t*) which was used to calculate the IACF was recorded after transmission through the sample. This transmitted intensity can be approximated to originate from the superposition of partial fields that are propagated along distinct paths through the sample.^23^ The summed electric field *E*(*t*) starts to fluctuate due to phase shifts which the partial electric fields accumulated along the paths through the sample, which turns the intensity *I*(*t*) sensitive to microscopic motions of scattering centers in the sample.^23^ The local packing fraction *ϕ*_loc_ fluctuates in time in fluidized granular media in addition to displacements of scattering centers.^12,14^ These density fluctuations can be taken into account by time-dependent amplitudes propagating along the distinct paths.^14^ The summed up instantaneous intensities of the paths yield then a total transmitted intensity *I*_*t*_(*t*), which fluctuates in time formally independent of the electric field *E*(*t*).^14^ The IACF ${\left\langle {I(t) I (t + \tau )} \right\rangle }$ consequently turns into the sum of the contributions ${\left\langle {I_t(t) I_t (t + \tau )} \right\rangle }$ and $\left| {\left\langle {E(t)E^ * (t + \tau )} \right\rangle } \right|^2$.^14^ The independence of *I*_t_(*t*) and *E*(*t*) is only formal, as the macroscopic density can be expected to influence the microscopic particle dynamics and by this the fluctuations of the electric field. The presence of temporal density fluctuations can be verified by monitoring the distribution of the intensity values, which obeys a Γ-distribution for normal distributed field values and becomes broader than a Γ-distribution with the emergence of local number density or local packing fraction fluctuations.^14,24^

We use the correlation of the total transmitted intensity $$ {\left\langle {I_t(t) I_t (t + \tau )} \right\rangle }$$ to grade the homogeneity of the fluidized state. The normalized amplitude at $$\tau = 0, \left\langle {I_{\mathrm{t}}(t)I_{\mathrm{t}}(t)} \right\rangle /\left\langle {I(t)} \right\rangle ^2 = 1 + \left\langle {{\mathrm{\Delta }}I_{\mathrm{t}}(t)^2} \right\rangle /\left\langle {I(t)} \right\rangle ^2 = :1 + \tilde V(\phi _{{\mathrm{loc}}})$$, as obtained from the hardware correlator, gives the normalized variance of the fluctuations of the total transmitted intensity. Here $$ \Delta {I_t(t)}$$ is the instantaneous difference among $$ {I_t(t)}$$ and the average $$ {\left\langle {I_t(t)} \right\rangle}$$, and $$ \langle {{\mathrm{\Delta }}I_{\mathrm{t}}(t)^2} \rangle$$ is the variance of $$ {I_t(t)}$$. A homogeneous state implies that the fluctuations of the local packing fraction become minimal. Consequently, the variance of fluctuations of the total transmitted intensity and the contribution of $$ {I_t(t)}$$ to the IACF will vanish in a homogeneous state.

The correlation of the electric field *E*(*t*) is employed to determine the degree of fluidization within the dense granular medium. The electric field will be normally distributed, since the field fluctuations arise from sums of phase-shifted waves traveling along different paths. The phase shifts can be assumed evenly distributed over intervals of 2*π*, and ${\left\langle E \right\rangle } = {\left\langle \Delta E \right\rangle } = 0$ and ${\left\langle E^2 \right\rangle } = {\left\langle \Delta E^2 \right\rangle }$, where Δ*E*(*t*) is the instantaneous difference among *E*(*t*) and ${\left\langle E(t) \right\rangle }$. The normalized amplitude of the contribution by the field autocorrelation $\tilde E: = \left| {\left\langle {E(t)E^ * (t)} \right\rangle } \right|^2/\left\langle {I(t)} \right\rangle ^2 = \left\langle {{\mathrm{\Delta }}E^2} \right\rangle ^2/\left\langle {I(t)} \right\rangle ^2$ will become unity in this case. Deviations of the amplitude from 1 indicate a deviation from the normal distribution of the field values. This could be due to a setup-dependent coherence-factor 1/*β*, as detection of *β* correlation areas (speckles) reduces the amplitude of the field correlation by a factor of 1/*β*.^25^ The previous experiments with this setup had shown a maximal amplitude of 0.3 throughout the experiments,^22^ thus the field correlation functions presented here are multiplied with an *β*-factor of 3 to account for the averaging over roughly three speckles. The amplitude is also reduced by a static component in the detected light^26^ and non-ergodicity of the sample.^25^ A reduction of the amplitude beyond the contribution by speckle averaging consequently indicates to which degree the sample became static or so slow that ergodicity is not reached anymore. It is not clear yet how exactly the field correlation obtained with 532 nm laser light relates to the motion of the particles, as translation and rotation of rough, inhomogeneous particles are probed on extremely short-length scales with respect to particle size. However, as the particles are the only mobile entities in the setup, field fluctuations must be related to the motion of the particles and the general conclusion can be drawn, whether the granular medium is homogeneously or only partially fluidized.

### Data availability

The data that support the findings of this study are available from the corresponding author upon request.
